# Prevalence of fibromyalgia among university students and its impact on their health-related quality of life: a survey-based study from Egypt

**DOI:** 10.1186/s12889-023-17329-5

**Published:** 2023-12-06

**Authors:** Samar Tharwat, Nourhan Ramadan Mosad, Kerolos Ebrahim Abdelmessih, Eman Moatamed, Mohamed Rihan, Nouran Osama, Norhan Sallam, Yara Elsayed

**Affiliations:** 1 Rheumatology & Immunology Unit, Department of Internal Medicine, Faculty of Medicine, Mansoura University, Mansoura, Dakahlia, Egypt; 2 Department of Internal Medicine, Faculty of Medicine, Horus University, New Damietta, Egypt; 3 Faculty of Medicine, Menoufia University, Shebin Elkom, Egypt

**Keywords:** Fibromyalgia, University students, Quality of life, SF36

## Abstract

**Background:**

University students are more likely to experience stress, anxiety, and depression. All these factors are regarded as psychological contributors to fibromyalgia syndrome (FMS).

**Aim:**

To investigate the prevalence and determinants of FMS among university students and its impact on their health-related quality of life (HRQoL).

**Methods:**

This online survey-based study involved 2146 university students who were recruited from various faculties at several Egyptian universities. The participants’ demographics, medical history, academic pursuits, and sleep data were collected. To identify the existence of FMS, the 2016 updates to the 2010/2011 FMS diagnostic criteria were used. Additionally, the participants completed the Short-Form Health Survey-36 (SF-36).

**Results:**

The mean age was 21.26 ± 2.015 years and 76% were females. Of 2146 students, 266 (12.4%) fulfilled the criteria of FMS. FMS group had a significantly lower age (*p* < 0.001) with predominant female gender (89.5% vs. 74.1%, *p* < 0.001), positive family history of FMS (8.6% vs. 3.7%, *p* < 0.001), previous history of traffic accident (10.2% vs. 6.8%, *p* = 0.045), lower level of physical activity (*p* = 0.002),higher time spent in study per week (*p* = 0.002), lower sleep time (*p* = 0.002), with frequent walk up (*p* < 0.001) and snoring (*p* < 0.001) during sleep. Regarding HRQoL, students with FMS had significantly lower scores than students without in all domains.

**Conclusion:**

FMS is prevalent among Egyptian university students and is linked to female gender, positive family history, lower levels of physical activity, and more time spent studying each week. FMS has a negative impact on HRQoL. Therefore, early detection and treatment are recommended.

## Introduction

Fibromyalgia syndrome (FMS) is a disorder characterized by chronic, widespread musculoskeletal pain. Muscle and joint stiffness, insomnia, fatigue, mood disorders, cognitive dysfunction, anxiety, and generalized sensitivity are the main manifestations of this disease [1]. FMS is estimated to affect 2–4% of the general population [2] and mainly affects women (61–90%) [3]. There are no specific tests that are pathognomonic or specific for FMS [4]. University students are more likely to experience stress, anxiety, and depression. All of these factors are regarded as psychological contributors to FMS [5].

The World Health Organization (WHO) defines quality of life (QoL) as a person’s “perception of their position in life in relation to their goals, expectations, standards, and concerns in the context of the culture and value systems in which they live.” [6]. By evaluating health-related QoL (HRQoL), it is possible to measure an individual’s health status and make standardized comparisons between different medical conditions [7]. The HRQoL of university students and the factors that may affect it have recently attracted a lot of attention [8].

The prevalence and correlates of health problems, including pain, among student samples have been assessed in several studies [9]. These studies demonstrate that pain is a widespread issue among university students and is associated with poorer psychological, social, and physical health [10]. According to a national health survey for higher education in Norway [11], 54% of university students report having chronic pain lasting more than three months at at least one pain site. Also, young adults under 22 had a 10.9% prevalence of chronic multisite pain [12]. Students who have chronic pain encounter more challenges than those who don’t [9] and have much poorer HRQoL [9]. However, the majority of prior research focused on pain prevalence rather than FMS and its impact on HRQoL in university students.

FMS among university students and its impact on their HRQoL are not commonly studied. So, the aim of this study was to investigate the prevalence and determinants of FMS among university students and its impact on their HRQoL.

## Materials and methods

### Study design and setting

This study was carried out as cross-sectional analytic research. It involved 2146 university students from several Egyptian universities. It was a survey-based study that required participants to fill out a self-administered online questionnaire generated on Google Forms. All students from several Egyptian universities who were over the age of 18 were eligible to participate in the study. From the 29th of January to the 28th of March 2022, the questionnaire was sent out at random to all potential participants via social media platforms (such as Facebook and WhatsApp).

Then they were directed to a webpage that explained the aim of the study and gave them instructions on how to fill out the questionnaire. Participants were guaranteed anonymity and data confidentiality. Anyone who accepted the study’s invitation was led to a Google Form. Answering and submitting all the questions is considered consent to participate in the study.

### Sample size calculation

The appropriate sample size was calculated using the online sample size calculator, RaoSoft®. The minimal sample size was 385 participants, based on an estimated population of 3 million students in Egyptian universities [13], a 50% expected response, a 5% margin of error, and a 95% confidence level.

### Ethical consideration

This study was carried out in accordance with the principles of the Helsinki Declaration [14], and the Institutional Research Board of Mansoura University’s Faculty of Medicine gave its approval to the study protocol (approval registration number: R.22.07.1758).

### Questionnaire structure

The researchers created the questionnaire after conducting a thorough review of the literature. The questionnaire, which was written in English, comprised multiple-choice questions. The questionnaire that was created underwent evaluation by a team consisting of four rheumatology staff members to provide their insights, conduct a thorough assessment, and validate the content of the questionnaire. Based on this premise, there were no additions made to the existing items. However, three items were removed and three were modified in terms of their wording. Following this, a pilot study was conducted with a sample of 20 university students who had diverse ages and backgrounds. The purpose of this pilot study was to assess the structure, clarity, and length of the questionnaire, as well as gather the participants’ overall perception of it. As a result of this pilot study, a few minor adjustments were made to the original questionnaire. The Cronbach’s alpha coefficient was employed to assess the internal consistency of the questionnaire. The obtained reliability coefficient of 0.84 suggests a good level of internal consistency. The statistical analysis of the main study did not include the data collected from students who had participated in the pilot study.

### Baseline demographic characteristics and clinical data

The questionnaire asked a number of questions about the participants’ age, gender, marital status, weight, height, level of physical activity, and whether they had a family history of FMS or a prior history of traffic accidents in order to elicit their demographic characteristics. Moreover, all participants were asked to complete a questionnaire on nine conditions to determine the presence of comorbidities. The following conditions were included in the list: irritable bowel syndrome, hypothyroidism, headache, anxious or depressed mood, low back pain, diabetes mellitus, hypertension, rheumatologic or autoimmune disorder.

### University study data

Additionally, one question was listed to determine the year of university education of the participants. The participants were also asked about their satisfaction with the study using one open-ended question that allowed students to express their opinion. A 5-point Likert scale was employed, with 1 being highly satisfied and 5 being highly unsatisfied. Responders were asked to determine the approximate number of hours they spent studying per week.

### Sleeping pattern

The time of sleep, average hours of sleep per day, frequent wakeups during sleep, any naps, snoring, sleeping pill intake, having sleep apnea, and if there are any changes in sleeping pattern during vacation or weekends were all asked about to assess the sleeping pattern among participants.

### Fibromyalgia diagnostic criteria and symptom severity scale (SSS)

The questionnaire was originally designed to answer questions about the presence of FMS and its associated manifestations. The 2016 revisions to the 2010/2011 FMS diagnostic criteria were employed [15]. These criteria were assessed to determine their suitability as diagnostic criteria in clinical settings as well as their effectiveness as classification criteria in research contexts [15].

It has 5 sections ;the first section is a measurement of the Widespread Pain Index (WPI), which is completed by identifying body areas where pain or tenderness was felt in the previous 7 days, with a total of 19 body areas identified as follows: shoulder girdle (left and right), upper arm (left and right), lower arm (left and right), hip (left and right), upper leg (left and right), lower leg (left and right), jaw (left and right), chest, abdomen, upper back, lower back, and neck. The WPI component has a maximum score of 19.

The second section comprises three questions about fatigue symptoms, cognitive problems, and unrefreshing sleep during the previous week, each of which is scored on a Likert scale from 0 (no problem) to 3 (severe problem) (severe: continuous, life-disturbing problems). The results were added together for a maximum score of 9.

The third section has three questions with a positive or negative response for the following somatic symptoms that occurred in the last six months: abdominal discomfort or cramps, depression, and headache, with a maximum score of 3. Sections 2 and 3 added together yield a Symptom Severity Score (SS), which has a range of 0–12. The fourth section inquiries about the presence of symptoms for more than 3 months, and the fifth section asks whether there is any other disorder that would explain the pain.

### Diagnosis of fibromyalgia

When all the following criteria were met, the subject was diagnosed with FMS:

1) Widespread pain index (WPI) score of 7 and symptom severity scale (SSS) score of 5 OR WPI 4–6 and SSS score of 9 OR WPI 4–6 and SSS score of 9.

2) There was generalized pain, which was defined as pain in at least four of the five body regions.

3) Symptoms had been present for at least 3 months at a similar degree.

4) FMS is a genuine diagnosis, regardless of other diagnoses. The presence of additional clinically significant disorders was not ruled out by a diagnosis of FMS.

### Short-form health survey-36 (SF-36)

The Short-Form Health Survey-36 (SF-36) [16] was employed to assess HRQoL. The SF-36 consists of 36 items assessing eight sub-dimensions: physical function (PF), social function (SF), role function–physical (RFP), role function–emotional (RFE), emotional well-being (EW), vitality (VT), bodily pain (BP), and general health perception (GHP). The SF-36 sub-dimensions were rated from 0 to 100, with a higher score indicating better health. Item scores were transformed to 0-100-point scales (0 = worst, 100 = best) using the SF-36 algorithm.

### Statistical analysis

For data analysis, the Statistical Package for Social Science (SPSS) program version 22 was utilized. Quantitative data was presented as means with standard deviations (SD) for parametric variables and medians (interquartile ranges) for nonparametric variables, while qualitative data was expressed as percentages and numbers. To determine the normality of the variable distribution, the Shapiro-Wilk test was used. For normally distributed data, the significance of differences between two groups was tested using the independent samples t test, and for non-parametric variables, the Mann-Whitney test was employed. For comparisons between qualitative variables, Chi-square or Fisher exact tests were utilized, as appropriate. Univariate and multivariate logistic regression analyses were used to identify factors linked with FMS in university students using the enter approach to assess the predictors of FMS. The goodness of fit for the model was tested using chi-square goodness of fit tests.

## Results

During the study period, 2215 individuals opened weblinks for the online survey, and 2146university students completed the online questionnaire. The mean age was 21.26 years, and women accounted for 76% of the participants. Most of the participants were single (96.5%), and only 53 (2.5%) had children. The mean body weight was 66.24 kg, and the mean height was 165.36 cm. Only 93 (4.3%) had a family history of FMS, and 154 (7.2%) reported a history of traffic accidents. Two hundred and sixty-six participants (12.4%) fulfilled the diagnostic criteria of FMS.

Demographic characteristics and clinical data of participants with and without FMS are illustrated in Table 1. There was no statistically significant difference between the 2 groups regarding marital status (*p* = 0.370), having children (*p* = 0.148), or smoking (*p* = 0.649). By comparing participants with FMS and those without, we found that the FMS group had a significantly lower age (*p* < 0.001) with a predominant female gender (89.5% vs. 74.1%, *p* < 0.001), a positive family history of FMS (8.6% vs. 3.7%, *p* < 0.001), previous history of traffic accidents (10.2% vs. 6.8%, *p* = 0.045),and a lower level of physical activity (*p* = 0.002). Although there was no significant difference between the two groups regarding the presence of diabetes mellitus, other associated conditions such as irritable bowel syndrome, hypothyroidism, headache, depressed mood, and low back pain were more prevalent in the FMS group.

**Table 1:** Baseline demographic characteristics and clinical data of the participants (n = 2146)

Variable mean ± SD, n (%)	Total (n = 2146)	Without fibromyalgia (n = 1880) 87.6%	With fibromyalgia (n = 266) 12.4%	*P*
Demographic characteristics				
Age, years	21.26 ± 2.015	21.33 ± 2.020	20.78 ± 1.918	< 0.001*
*Gender*				
Male Female	515 (24) 1631 (76)	487 (25.9) 1393 (74.1)	28 (10.5) 238 (89.5)	< 0.001*
*Marital status*				
Single Married Widow Divorced	2071 (96.5) 69 (3.2) 1 (0) 5 (0.2)	1819 (96.8) 56 (3) 1 (0.1) 4 (0.2)	252 (94.7) 13 (4.9) 0 1 (0.4)	0.370
Having children	53 (2.5)	43 (2.3)	10 (3.8)	0.148
*Special habit*				
Smoking Alcohol	46 (2.1) 7 (0.3)	42 (2.2) 7 (0.4)	4 (1.5) 0	0.649 1
*Anthropometric measures*				
Weight, Kg Height, Cm Body mass index (Kg/m^2^)	66.24 ± 13.01 165.36 ± 8.63 24.18 ± 4.03	66.25 ± 12.86 165.59 ± 8.72 24.11 ± 3.92	66.11 ± 14.05 163.69 ± 7.78 24.62 ± 4.7	0.873 0.001* 0.055
*Physical activity level*				
Low Moderate High	627 (29.2) 1372 (63.9) 147 (6.8)	525 (27.9) 1223 (65.1) 132 (7)	102 (38.3) 149 (56) 15 (5.6)	0.002*
Family history for fibromyalgia	93 (4.3)	70 (3.7)	23 (8.6)	< 0.001*
Traffic accident	154 (7.2)	127 (6.8)	27 (10.2)	0.045*
Clinical data				
Irritable bowel syndrome	764 (35.6)	602 (32)	162 (60.9)	< 0.001*
Hypothyroidism	90 (4.2)	64 (3.4)	26 (9.8)	< 0.001*
Headache	1252 (58.3)	1013 (53.9)	239 (89.8)	< 0.001*
Anxious mood	1364 (63.6)	1124 (59.8)	240 (90.2)	< 0.001*
Depressed mood	793 (37)	614 (32.7)	179 (67.3)	< 0.001*
Low back pain	947 (44.1)	724 (38.5)	223 (83.8)	< 0.001*
Diabetes mellitus	30 (1.4)	23 (1.2)	7 (2.6)	0.067
Hypertension	58 (2.7)	38 (2)	20 (7.5)	< 0.001*
Rheumatologic or autoimmune disorder	110 (5.1)	77 (4.1)	33 (12.4)	< 0.001*
Others	113 (5.3)	87 (4.6)	26 (9.8)	< 0.001*

* *P* < 0.05

**Fig. 1:**
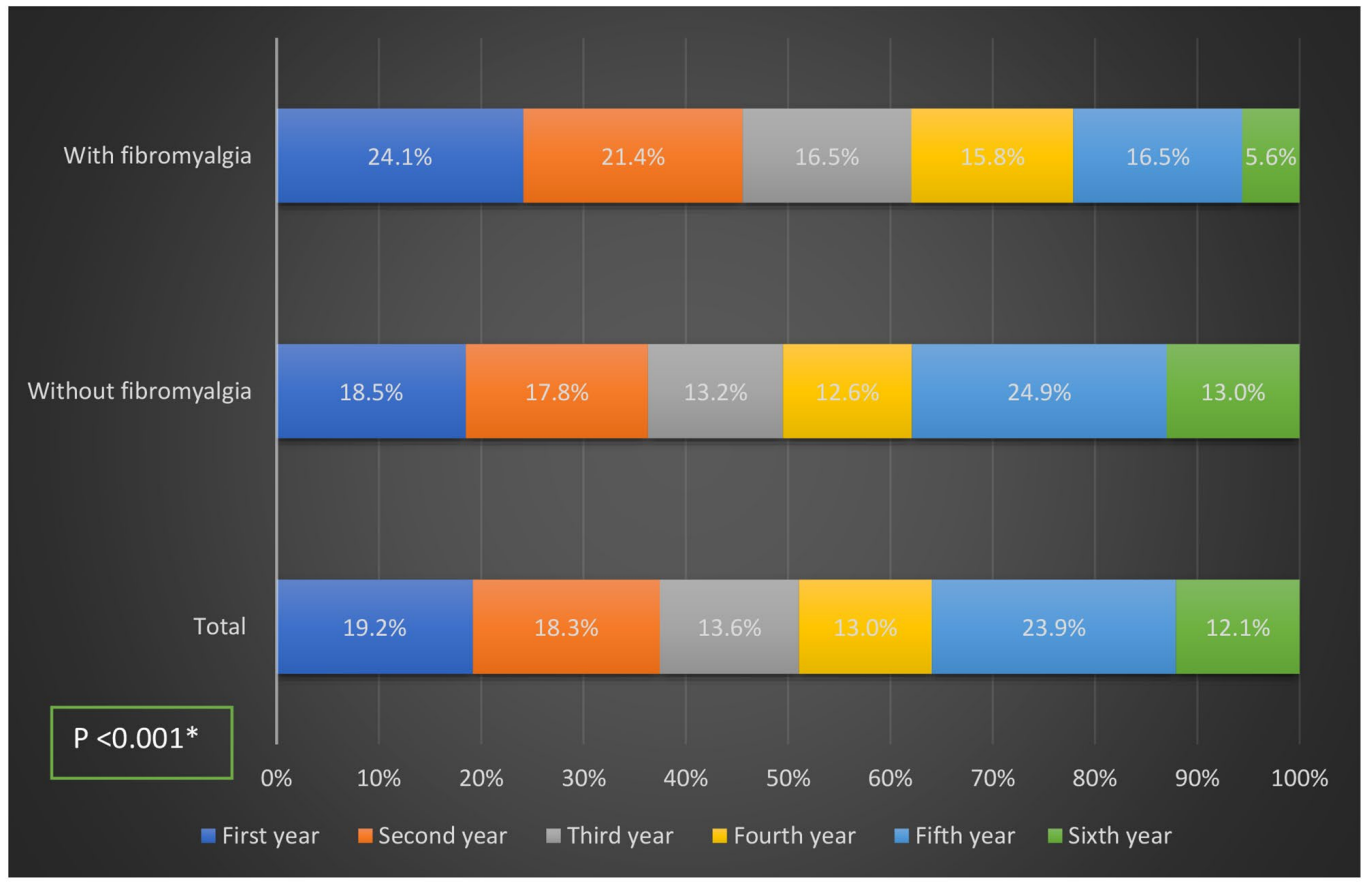
Year of university education of the students with and without fibromyalgia

As shown in Fig. 1, the participants came from different academic levels:19.2% in the first year,18.3% in the second,13.6% in the third,13% in the fourth ,23.9% in the fifth and 12.1% in the sixth. FMS was more prevalent among university students in the early years of university education.

**Fig. 2:**
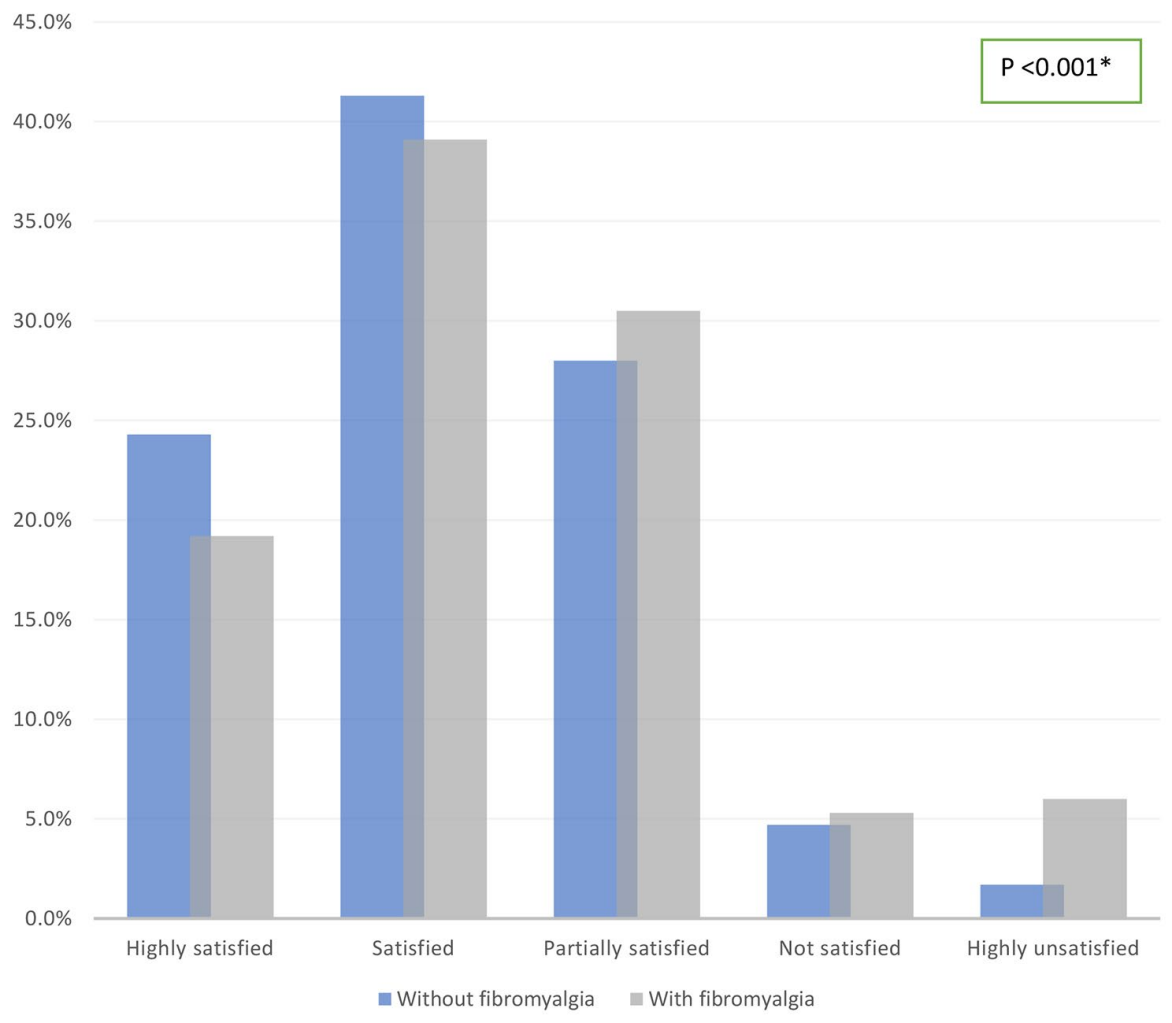
Satisfaction with the study among participants with and without fibromyalgia

Figure 2 illustrates the satisfaction with the study among participants with and without FMS. Although about 40% of the participants reported that they were satisfied with the study, there was a statistically significant difference between the two groups regarding their degree of satisfaction. About 24% of the non-FMS group were highly satisfied versus, 19% in the FMS group. Also,1.7% the non-FMS group were highly unsatisfied compared to6% of the FMS group (*p* < 0.001).

**Fig. 3:**
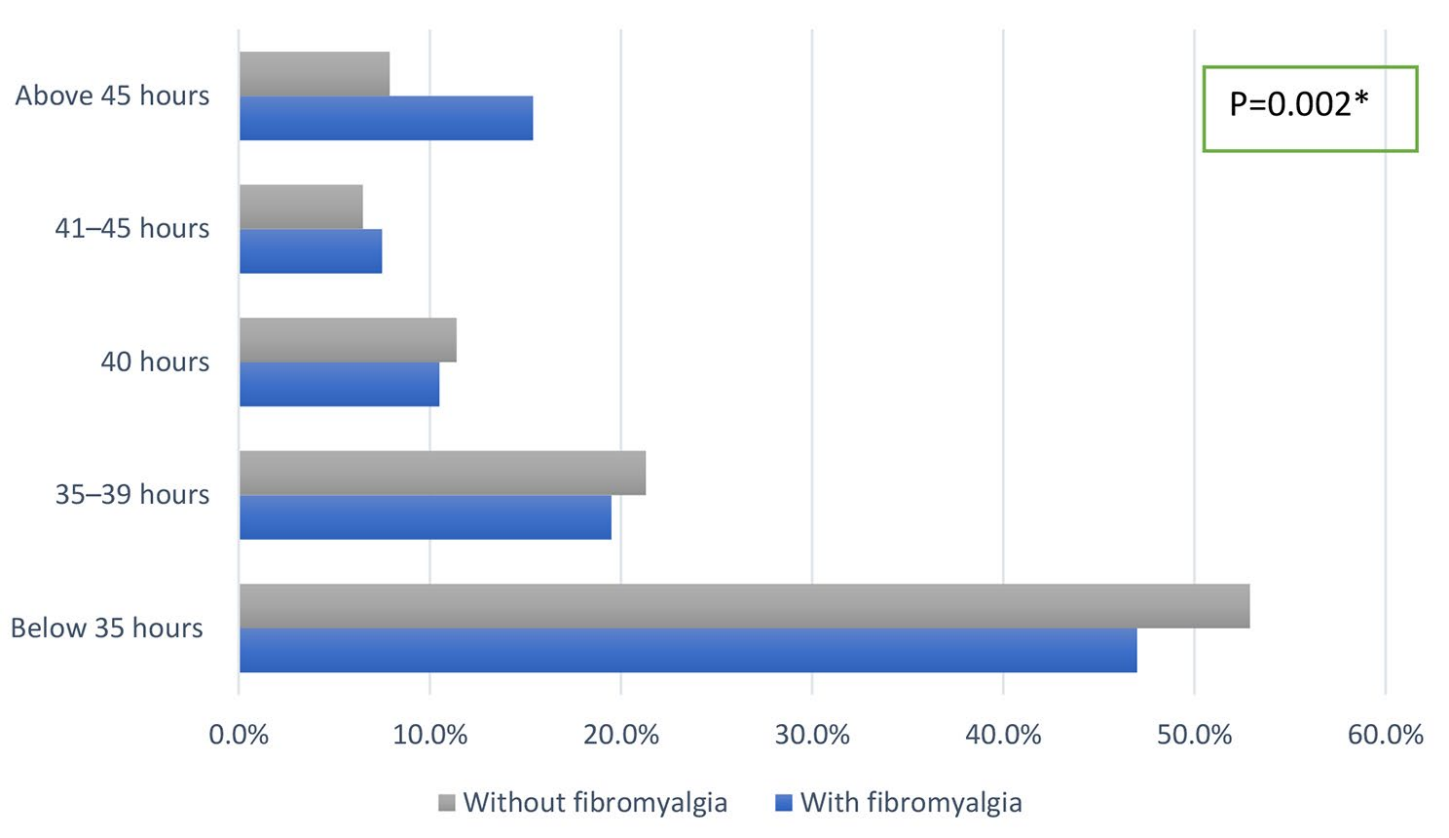
Number of hours in study per week among participants with and without fibromyalgia

When we evaluated the number of hours spent in study per week, we found that the FMS group had a higher time spent in study per week in comparison to the non-FMS group (*p* = 0.002) as shown in Fig. 3.

When considering the relationship between the sleeping pattern and FMS, it is observed that university students with FMS had lower sleep time (*p* = 0.002), with frequent walk-ups (*p* < 0.001) and snoring (*p* < 0.001) during sleep, as shown in Table 2.

**Table 2:** Sleeping pattern of the participants (n = 2146)

Variable mean ± SD, n (%)	Total (n = 2146)	Without fibromyalgia (n = 1880) 87.6%	With fibromyalgia (n = 266) 12.4%	*P*
*Sleep time*				
Before 12 AM After 12 AM	601 (28) 1545 (72)	531 (28.2) 1349 (71.8)	70 (26.3) 196 (73.7)	0.512
Average hours of sleep per day	7.71 ± 1.67	7.75 ± 1.63	7.4 ± 1.88	0.002*
Frequently wake up during sleep	660 (30.8)	522 (27.8)	138 (51.9)	< 0.001*
Take naps during the day	622 (29)	542 (28.8)	80 (30.1)	0.675
Snore during sleep	95 (4.4)	68 (3.6)	27 (10.2)	< 0.001*
Take sleeping pills	55 (2.6)	40 (2.1)	15 (5.6)	0.001*
Sleep apnea or other sleeping disorders	324 (15.1)	232 (12.3)	92 (34.6)	< 0.001*
*Sleeping pattern changes during vacation or weekends*				
No change Sleep earlier Sleep later	441 (20.5) 172 (8) 1533 (71.4)	406 (21.6) 147 (7.8) 1327 (70.6)	35 (13.2) 25 (9.4) 206 (77.4)	0.006*

* *P* < 0.05

**Fig. 4:**
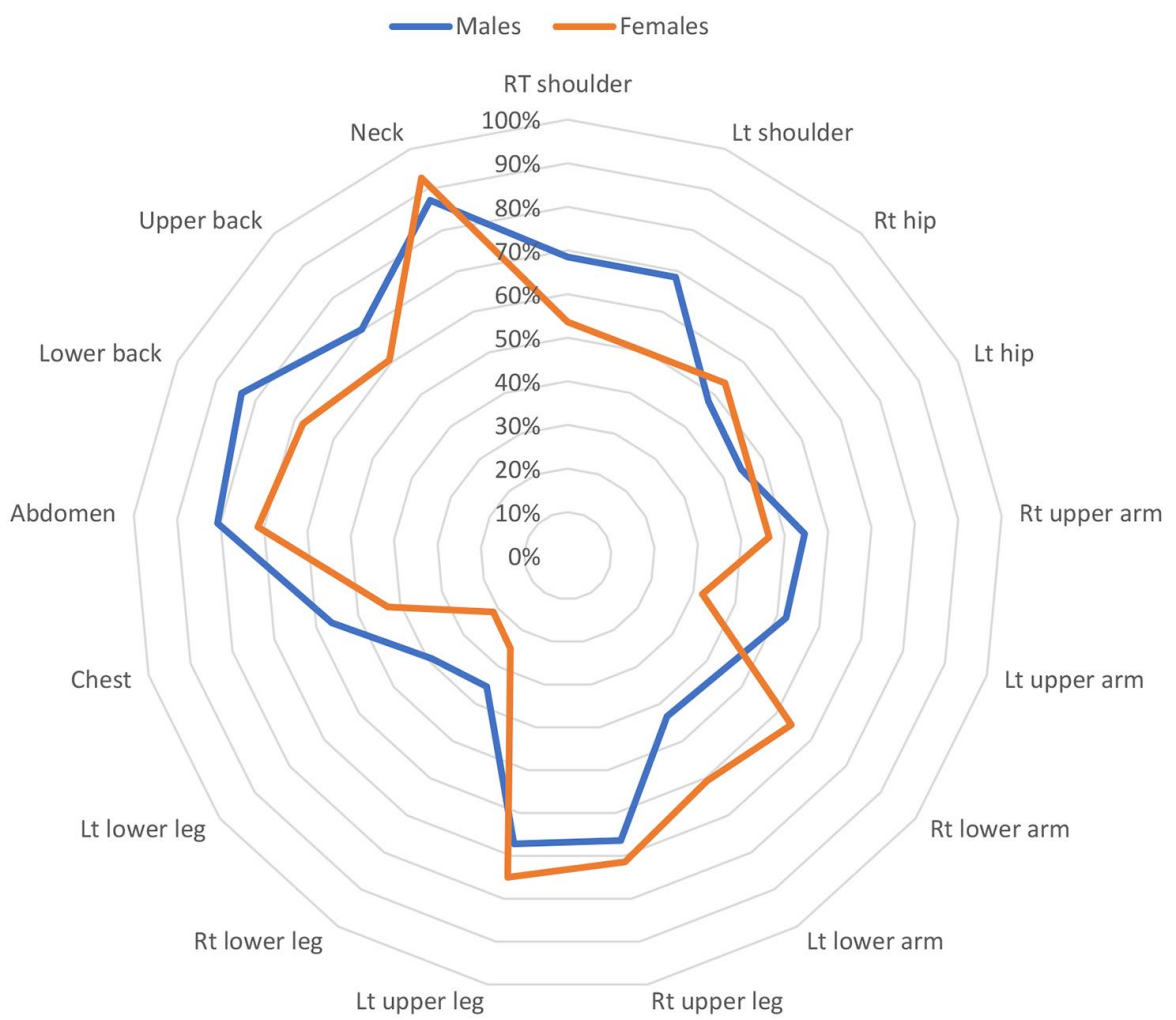
Sites of distribution of widespread pain in subjects with fibromyalgia (n = 266) Lt: left, Rt: right

The sites of distribution of widespread pain in subjects with FMS are illustrated in Fig. 4. According to the body regions, the maximum number of female participants with FMS reported pain in the neck (93%), followed by the left and right upper legs (75% and71% respectively), while most male participants with FMS reported pain in the neck (87%), followed by the lower back (84%), and abdomen (81%).

**Fig. 5:**
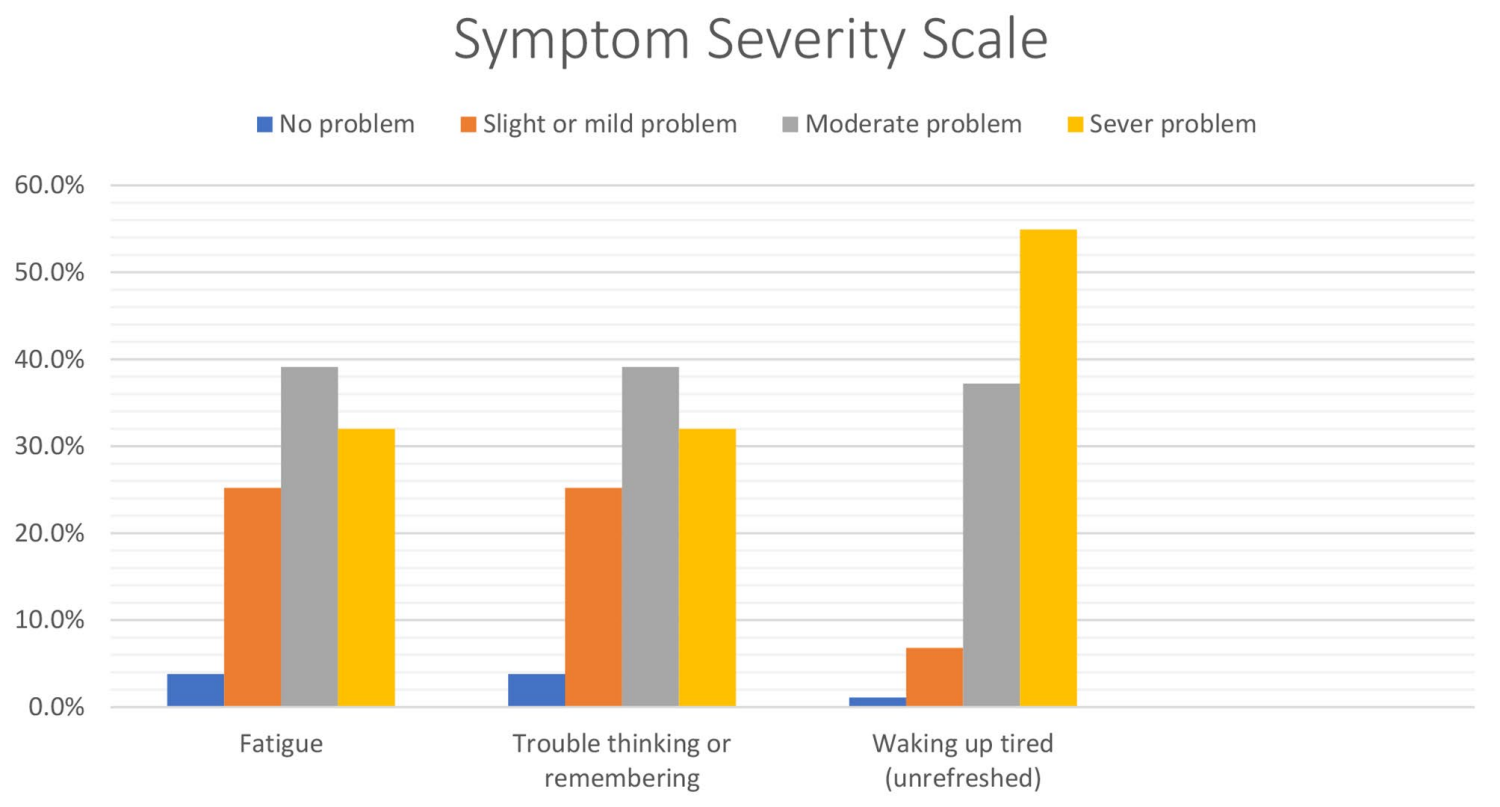
Symptom severity scale in subjects with fibromyalgia (n = 266)

The symptom severity scale is shown in Fig. 5, illustrating the degree of severity of fatigue, trouble thinking, and walking up tired in subjects with FMS.

The study employed the scores of SF-36 domains according to the presence or absence of FMS. University students with FMS had significantly lower scores than did those without in all HRQOL domains, as described in Table 3. The variables linked with FMS among university students are outlined in Table 4.

**Table 3:** Health related quality of life among university students with and without fibromyalgia

Variable median (IQR)	Total (n = 2146)	Without fibromyalgia (n = 1880) 87.6%	With fibromyalgia (n = 266) 12.4%	*P*
Physical functioning	80 (45)	80 (45)	65 (35)	< 0.001*
Role limitations due to physical health	25 (100)	25 (100)	0 (25)	< 0.001*
Role limitations due to emotional problems	33.33 (100)	33.33 (100)	0 (0)	< 0.001*
Energy/fatigue	50 (20)	50 (20)	30 (25)	< 0.001*
Emotional well-being	52 (24)	52 (28)	36 (21)	< 0.001*
Social functioning	62.5 (25)	62.5 (37.5)	50 (37.5)	< 0.001*
Pain	67.5 (45)	77.5 (35)	45 (22.5)	< 0.001*
General health	50 (15)	52.5 (15)	40 (15)	< 0.001*
SF-36 PCS	55.63 (31.25)	58.75 (31.25)	40.31 (15.63)	< 0.001*
SF-36 MCS	47 (35.73)	50.56 (35.73)	30.63 (20.31)	< 0.001*

* *P* < 0.05

SF-36: 36-Item Short Form; PCS: physical component summary; MCS: mental component summary

**Table 4:** Univariate and multivariate logistic regression analyses of factors associated with fibromyalgia in university students (n = 2146)

Variable	Univariate analysis			Multivariate analysis		
	OR	95% CI	*p*	OR	95% CI	*p*
Age	0.870	0.814–0.930	< 0.001*			
Female gender	2.972	1.982–4.455	< 0.001*	2.563	1.668–3.940	< 0.001*
Smoking	0.668	0.238–1.878	0.444			
Physical activity level	0.683	0.54–0.861	0.001*	0.664	0.517–0.853	0.001*
Family history for fibromyalgia	2.447	1.500-3.994	< 0.001*	2.102	1.240–3.565	0.006*
Traffic accident	1.558	1.006–2.411	0.047*			
Academic year	0.847	0.787–0.911	< 0.001*			
Number of hours in study per week	1.174	1.072–1.287	0.001*	1.169	1.059–1.289	0.002*
Average hours of sleep per day	0.876	0.807–0.951	0.002*	0.975	0.828–0.975	0.010*
Frequently wake up during sleep	2.805	2.161–3.641	< 0.001*	2.503	1.399–2.503	0.001*
Snore during sleep	3.010	1.889–4.796	< 0.001	3.527	1.245–3.527	0.005*
Take sleeping pills	2.749	1.497–5.049	0.001			
Sleep apnea or other sleeping disorders	3.756	2.817–5.007	< 0.001	2.199	1.590–3.041	< 0.001*

*p < 0.05

## Discussion

To the best of our knowledge, this is the first detailed study about FMS among Egyptian University students that encountered the 2016 revisions to the 2010/2011 FMS diagnostic criteria [15]. The study includes students from several Egyptian universities and provides data about the relationshipbetween FMs and HRQoL among university students.

In the current study, we found that 12.4% of Egyptian university students had FMS. The FMS group was much younger, primarily female, had a positive family history of the condition, had experienced traffic accidents in the past, and engaged in less physical activity. Also, irritable bowel syndrome, hypothyroidism, headaches, low mood, and back pain were more common in the FMS group. The FMS group spent more time studying each week and had lesssleep time. FMS was associated with a reduction in HRQoL.

FMS is a stress-related disorder; environmental stressors, including physical and psychological stressors,are common drivers of FMS [17]. University students make up a special group since they face a wide range of stressors. Due to the differences in the educational system in terms of new teaching methods, academic requirements, types of relationships between students and faculty, and even relationships among students themselves. The transition of students from the school environment to the university environment could cause psychological, academic, and social shocks [18].

We employed 2016 FMS criteria in this study. These criteria continue to be highly valuable for clinical and epidemiological research endeavors that necessitate the identification of individuals with FMS [19]. According to Ahmed et al. [20], the 2016 revised criteria have the potential to identify distinct subgroups of individuals with fibromyalgia (FMS) who experience persistent, widespread pain. These criteria aim to enhance the effectiveness of diagnosing FM based on symptoms by excluding patients with regional pain syndromes [21].

In this study, the prevalence of FMS among university students was 12.4%. Several studies have estimated that between 2 and 9% of general populations have FMS [22, 23]. Results of a recent meta-analysis of 65 studies, which included 3,609,810 subjects from both the general population and specific groups, revealed that 1.78%, particularly women, suffer from FMS [24]. However, little research has been conducted among university students. In a cross-sectional study conducted on 293 members of the Saudi Pharmaceutical Society using an online questionnaire, 52% of the individuals reported having generalized body pain [25]. In another study in which 450 medical students were involved,43 (9.6%) were found to have FMS overall [26].

In this study, there is a significant gender disparity linked to FMS diagnosis, with the majority of those affected being women. This finding is in line with a large body of literature that indicates FMS is far more common in females, with the male-female ratio ranging from 1:2 to 1:9 [1, 27–29]. The disparities between the genders could be explained by biological characteristics connected to endogenous pain-relieving mechanisms or the influence of gonadal hormones [30].

The results of this study indicate that the FMS group has a statistically significant positive family history of FMS. It became clear early on in the study of FMS that family aggregation is crucial to the epidemiology of this condition. Parents and siblings of FMS patients had a higher frequency of either FMS or muscular tenderness [31]. The odds ratio of FMS in a relative of a proband with FMS compared to a proband with rheumatoid arthritis is 8.5, indicating that FMS is significantly aggregated in families. Additionally, tenderness is substantially aggregated. These data indicate a significant contribution of genetic factors to the pathogenesis of FMS [32].

In the present study, it is noteworthy that the FMS group reported reduced levels of physical activity. The general public’s health status has been inextricably linked to sedentary time [33]. Sedentary lifestyles have been linked to an increased risk of FMS [34]. It is well known that FMS patients exhibit lower levels of physical activity than their counterparts [35].

The subjective component of HRQoL, known as life satisfaction, refers to a person’s perceptions about their functioning and circumstances [36]. In turn, it is generally believed that an adult’s psychological development and well-being depend on their level of life satisfaction [37]. FMS and psychological distress are strongly correlated [38]. In the present study ,we found that study satisfaction is significantly lower among the FMS group, while time spent in the study per week is significantly higher. University students, particularly those studying the medical sciences, are more likely to experience depression, which is linked to their satisfaction with their academic work [39].

Another important finding in this study was that the FMS group had a short sleep time, with frequent walk-ups and snoring during sleep. In healthy people, sleep restriction can lead to FMS symptoms including myalgia, tenderness, and exhaustion, indicating that sleep dysfunction may not just be a consequence of pain but also pathogenic [4]. In a meta-analysis of 25 case-controlled studies with 2086 individuals, sleep was investigated using polysomnography and the Pittsburgh Sleep Quality Index, the results revealed that FMS patients had shorter sleep durations, lighter sleep, and longer wake times after starting to sleep [40]. Also, poor sleep is substantially and dose-dependently associated with symptom severity in the FMS population [41]. Sleep deprivation affects descending pain-inhibition pathways, which are critical for managing and coping with pain [4].

HRQoL is a growing problem in FMS. In the current study, when comparing the scores of HRQoL domains according to the presence or absence of FMS, students with FMS had significantly lower scores than those without. Previous studies have compared FMS patients with other subjects and found that FMS patients have a worse health status than patients with other chronic diseases, such as osteoarthritis, rheumatoid arthritis, systemic lupus erythematosus, myocardial infarction, chronic obstructive pulmonary disease, congestive heart failure, hypertension, and diabetes, as well as healthy control subjects [42–47]. Patients with chronic pain have difficulty performing daily routine activities. Due to these challenges, they are less able to engage in social interactions [48].

Finally, several important limitations need to be considered. First, the major point is that there was no laboratory testing conducted and that FMS was diagnosed using a self-administrated questionnaire. Second, due to the fact that all assessments were based on self-reports, collection bias was inevitable. Third, many different medical disorders can contribute to some FMS symptoms, such as fatigue and sleeplessness. Due to the nature of the study, these conditions could not be ruled out. Finally, it would have been preferable for the research to focus only on the female population, given the higher prevalence of this disease among females.

## Conclusion

Regardless of these limitations, the research findings provide evidence regarding the high prevalence of FMS among university students and its negative impact on their HRQoL. Early detection of FMS and early interventions may be the most effective methods to prevent problems that may arise later.

Our findings point to the necessity for further research to develop comprehensive theoretical models for comprehending the mechanisms underlying the HRQoL of university students with a diagnosis of FMS. It is necessary to conduct long-term studies with a variety of participants and disease groups, with a special emphasis on the use of comprehensive measures and objective health status evaluation.

## Data Availability

The datasets generated during and/or analysed during the current study are available from the corresponding author on reasonable request.

## Abbreviations

FMS: Fibromyalgia syndrome
HRQoL: Health-related quality of life
SF-36: Short-Form Health Survey-36
WHO: World Health Organization
